# Disparities in immune and targeted therapy utilization for older US patients with metastatic renal cell carcinoma

**DOI:** 10.1093/jncics/pkad036

**Published:** 2023-05-18

**Authors:** Ryan D Chow, Jessica B Long, Sirad Hassan, Stephanie B Wheeler, Lisa P Spees, Michael S Leapman, Michael E Hurwitz, Hannah D McManus, Cary P Gross, Michaela A Dinan

**Affiliations:** Yale School of Medicine, New Haven, CT, USA; Yale Cancer Outcomes, Public Policy, and Effectiveness Research Center, New Haven, CT, USA; Yale Cancer Outcomes, Public Policy, and Effectiveness Research Center, New Haven, CT, USA; Department of Health Policy and Management, Gillings School of Global Public Health, University of North Carolina at Chapel Hill (UNC-CH), Chapel Hill, NC, USA; Lineberger Comprehensive Cancer Center, UNC-CH, Chapel Hill, NC, USA; Department of Health Policy and Management, Gillings School of Global Public Health, University of North Carolina at Chapel Hill (UNC-CH), Chapel Hill, NC, USA; Lineberger Comprehensive Cancer Center, UNC-CH, Chapel Hill, NC, USA; Yale Cancer Outcomes, Public Policy, and Effectiveness Research Center, New Haven, CT, USA; Department of Urology, Yale School of Medicine, New Haven, CT, USA; Department of Internal Medicine, Yale School of Medicine, New Haven, CT, USA; Department of Medicine, Duke University School of Medicine, Durham, NC, USA; Yale Cancer Outcomes, Public Policy, and Effectiveness Research Center, New Haven, CT, USA; Department of Internal Medicine, Yale School of Medicine, New Haven, CT, USA; Yale Cancer Outcomes, Public Policy, and Effectiveness Research Center, New Haven, CT, USA; Department of Chronic Disease Epidemiology, Yale School of Public Health, New Haven, CT, USA

## Abstract

Disparities in metastatic renal cell carcinoma (mRCC) outcomes persist in the era of oral anticancer agents (OAAs) and immunotherapies (IOs). We examined variation in the utilization of mRCC systemic therapies among US Medicare beneficiaries from 2015 to 2019. Logistic regression models evaluated the association between therapy receipt and demographic covariates including patient race, ethnicity, and sex. In total, 15 407 patients met study criteria. After multivariable adjustment, non-Hispanic Black race and ethnicity was associated with reduced IO (adjusted relative risk ratio [aRRR] = 0.76, 95% confidence interval [CI] = 0.61 to 0.95; *P *=* *.015) and OAA receipt (aRRR = 0.76, 95% CI = 0.64 to 0.90; *P *=* *.002) compared with non-Hispanic White race and ethnicity. Female sex was associated with reduced IO (aRRR = 0.73, 95% CI = 0.66 to 0.81; *P *<* *.001) and OAA receipt (aRRR = 0.74, 95% CI = 0.68 to 0.81; *P *<* *.001) compared with male sex. Thus, disparities by race, ethnicity, and sex were observed in mRCC systemic therapy utilization for Medicare beneficiaries from 2015 to 2019.

Racial disparities in survival for patients with renal cell carcinoma (RCC) have been well-documented (1-4). Notably, these disparities persisted in the era of targeted therapies and oral anticancer agents (OAAs) (5). Prior studies have highlighted potential drivers of these racial disparities, including inequitable access to health care (6,7), differences in disease biology (8,9), and variable adherence to guideline-based treatment (10). Sex- or gender-related disparities in RCC have also been described (11), with studies reporting differences in tumor biology at the time of presentation (9,12,13) and varying rates of treatment utilization (10,14,15) between males and females.

We previously observed that from 2007 to 2015, race and ethnicity was not statistically significantly associated with OAA utilization for metastatic RCC (mRCC) (16). Given the shifting landscape of mRCC treatment following the introduction of checkpoint inhibitor immunotherapies (IOs) (17-19), a key knowledge gap has arisen as to whether disparities by race, ethnicity, or sex have since emerged. Whereas OAAs can be readily self-administered in a non–health-care setting, IOs involve intravenous infusions by trained personnel, potentially exacerbating existing inequities in health-care access. Quantitatively identifying specific patient groups with reduced therapy utilization is a key first step toward designing interventions to equitably broaden the impact of new cancer therapies.

Here, we evaluated the utilization of mRCC systemic therapy in older US patients from 2015 to 2019, capturing a unique time window that encompasses the advent of IO for mRCC treatment but largely predates the use of combination of concurrent OAA and IO regimens (20,21). We investigated how the approval of IO reshaped the mRCC systemic therapy landscape, specifically interrogating whether these shifts have differentially impacted patients of diverse race and ethnicities and sexes.

We conducted a retrospective cohort study of Medicare beneficiaries aged 66 years and older diagnosed with mRCC from 2015 through 2019, who were enrolled in fee-for-service Medicare parts A, B, and D from 1 year prior through 1 year after presumed diagnosis or until death (Supplementary Figure 1, A and B, and Supplementary Methods, available online). We queried claims from 2014 to 2020, identifying receipt of IOs, OAAs, or other systemic therapies in the 2 months before through 1 year after diagnosis and categorized patients by the first therapy received. Race and ethnicity (from the Research Triangle Institute Race Code variable) was categorized as American Indian and Native Alaskan or Other (combined because of small sample sizes precluding the reporting of data); Asian and Pacific Islander; Hispanic, non-Hispanic Black; or non-Hispanic White.

We identified 15 407 patients who were diagnosed with mRCC between 2015 and 2019 and met study criteria (Supplementary Tables 1 and 2, available online). The proportion of patients receiving any systemic therapy increased from 51.2% in 2015 to 59.2% in 2019 (*P *<* *.001) (Figure 1, A). The use of IO as initial therapy increased from 4.1% of patients in 2015 to 37.2% in 2019 (*P *<* *.001), whereas OAA use decreased from 31.2% in 2015 to 10.8% in 2019 (*P *<* *.001). IOs first surpassed OAAs as initial therapy in 2018. In 2015, 44.7% of non-Hispanic Black patients received systemic therapy compared with 51.3% of non-Hispanic White patients, corresponding to a differential of -6.6% (*P *=* *.08) (Figure 1, B). In 2019, this treatment differential nearly doubled to -12.4% (*P *<* *.001). Non-Hispanic Black patients received IOs at lower rates than non-Hispanic White patients (Figure 1, C) and received OAAs at lower rates than patients of other race and ethnicities (Figure 1, D).

**Figure 1.**
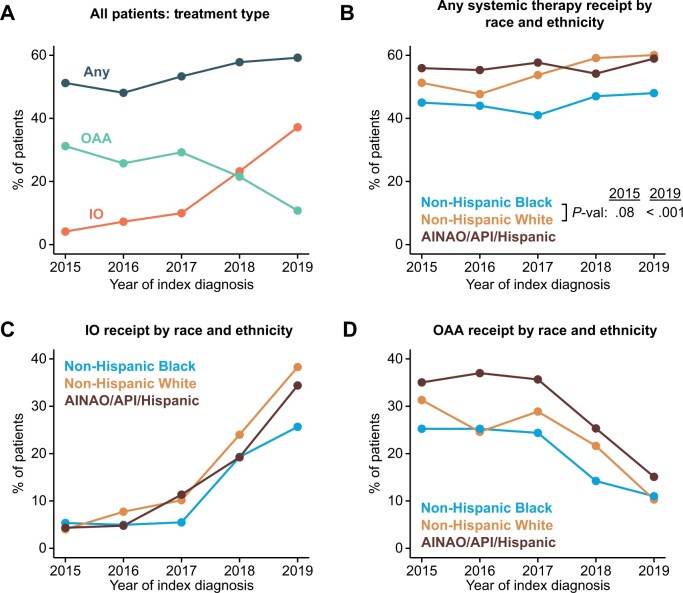
The evolution of initial systemic treatment for mRCC from 2015 to 2019 across racial and ethnic groups. **A)** Trends in initial systemic treatment from 2015 to 2019, categorizing patients by receipt of any systemic therapy, immunotherapy (IO), or oral anticancer agents (OAA). Data are expressed as unadjusted percentages of patients diagnosed with mRCC in a given year. **B-D)** Trends in utilization of any systemic therapy **(B)**, IO **(C)**, and OAA **(D)** from 2015 to 2019, stratified by racial and ethnic group. Because of small sample sizes, multiple races and ethnicities were combined for visualization, as indicated. Data are expressed as unadjusted percentages of patients diagnosed with mRCC in a given year. AINAO = American Indian, Native Alaskan, or all other; API = Asian and Pacific Islander; mRCC = metastatic RCC.

We next constructed logistic regression models with the initial systemic therapy received as the dependent variable, and several socioeconomic and demographic factors including race, ethnicity, and sex as independent variables. These covariates were selected through a joint consideration of clinical guidance, prior research, and data availability. After multivariable adjustment, non-Hispanic Black patients had lower rates of receiving any treatment (adjusted odds ratio [aOR] = 0.79, 95% confidence interval [CI] = 0.69 to 0.90; *P *=* *.001) (Figure 2, A). In particular, non-Hispanic Black patients had lower IO (adjusted relative risk ratio [aRRR] = 0.76, 95% CI = 0.61 to 0.95; *P *=* *.015) and OAA (aRRR = 0.76, 95% CI = 0.64 to 0.90; *P *=* *.002) (Figure 2, B and C) receipt. Hispanic patients had increased receipt of any treatment (aOR = 1.28, 95% CI = 1.09 to 1.50; *P *=* *.002), specifically OAAs (aRRR = 1.49, 95% CI = 1.24 to 1.79; *P *<* *.001) but similar rates of IO receipt (aRRR = 1.08, 95% CI = 0.83 to 1.39; *P *=* *.57). Female sex was associated with reduced receipt of any treatment (aOR = 0.78, 95% CI = 0.73 to 0.84; *P *<* *.001) including IO (aRRR = 0.73, 95% CI = 0.66 to 0.81; *P *<* *.001) and OAA (aRRR = 0.74, 95% CI = 0.68 to 0.81; *P *<* *.001) receipt. We further evaluated potential interactions between race and ethnicity and sex. Overall, non-Hispanic Black males, non-Hispanic White females, and non-Hispanic Black females had lower treatment rates compared with non-Hispanic White males, with the most pronounced reduction in therapy utilization for non-Hispanic Black females (Supplementary Figure 1, C, available online).

**Figure 2.**
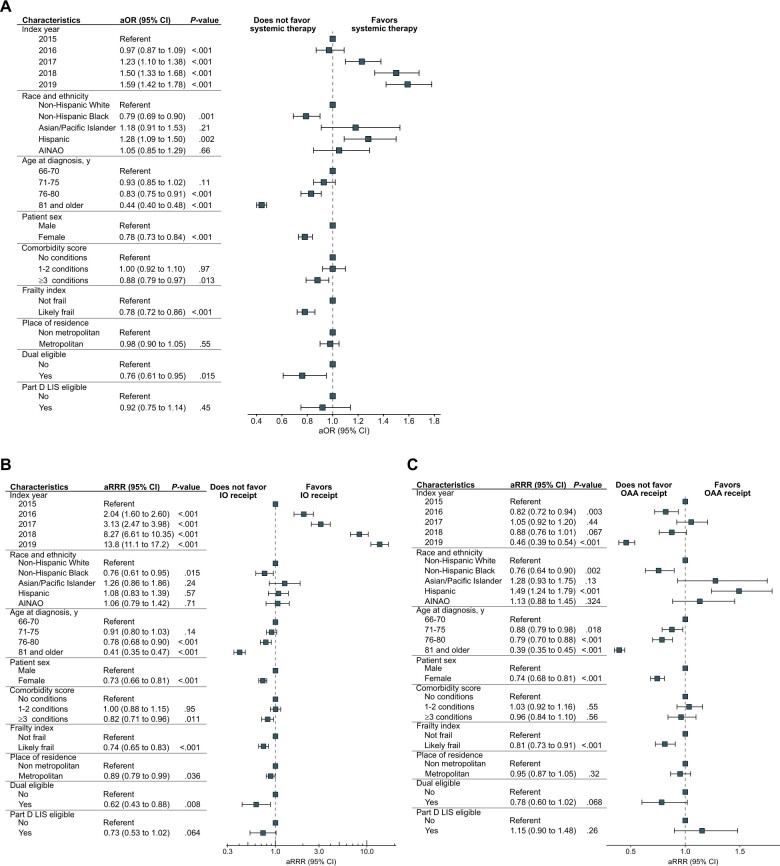
Factors associated with receipt of systemic therapy for metastatic RCC. **A-C).** Forest plots detailing the association between several demographic characteristics and receipt of any systemic therapy **(A)**, IOs **(B)**, or OAAs (C). Estimates for **panel A** are from a multivariable regression model with receipt of any systemic therapy as the outcome and the indicated covariates as predictors, expressed as adjusted odds ratios (aORs) with 95% confidence intervals (CIs). Estimates for **panels B-C** are similarly derived from a multinomial regression model with the specific first-line systemic treatment received as the outcome, expressed as relative risk ratios (aRRRs). AINAO = American Indian, Native Alaskan, and all other; IO = inhibitor immunotherapy; LIS = low-income subsidy; OAA = oral anticancer agent.

In this analysis of systemic therapy utilization for newly diagnosed mRCC from 2015 to 2019 among Medicare beneficiaries, we observed statistically significant disparities by race, ethnicity, and sex in IO and OAA receipt. The proportion of patients receiving systemic therapy for mRCC increased from 2015 to 2019, suggesting that the introduction of checkpoint IO shifted the equilibrium toward increased treatment utilization. However, these advances in therapy did not equally impact patients across racial and ethnic groups, as non-Hispanic Black race and ethnicity was independently associated with lower rates of OAA and IO receipt. Of particular concern, racial disparities in mRCC systemic therapy utilization appeared to have worsened from 2015 to 2019—a divergence from our prior work that found no significant association between race and ethnicity with OAA receipt from 2007 to 2015 (16).

Female sex was also associated with lower rates of OAA and IO receipt. Similar associations between sex and receipt of systemic therapy have previously been reported in patients with mRCC (22), bladder cancer (23), and lung cancer (24). Of note, the intersection of race and ethnicity and sex cooperatively impacted mRCC treatment utilization, with non-Hispanic Black females having among the lowest rates of therapy receipt.

A limitation of this claims-based study is that we could not account for differences in tumor characteristics, such as histological features, detailed staging, or other risk-modifying features, as well as individual-level indications or contraindications for certain treatments. Other limitations include potential errors in *International Classification of Diseases*–10 code capturing in claims data, the inability of Healthcare Common Procedure Coding System codes to capture inpatient therapies, and the absence of other socioeconomic variables that were not represented in the data.

This study marks only the first step. Future research is needed to understand 1) the underlying social determinants of health driving these disparities, such as systemic racism, provider factors, and inequities in health-care access; 2) whether such disparities are present across different health-care systems; 3) the impact of these disparities on disease control and survival; and 4) root-cause analyses to inform interventions or policies that will mitigate these disparities.

## Supplementary Material

pkad036_Supplementary_Data

## Data Availability

The data used in this study were obtained through the Chronic Conditions Data Warehouse (https://www2.ccwdata.org/web/guest/medicare-data).
