# Supplementary material for: Disparities in immune and targeted therapy utilization for older US patients with metastatic renal cell carcinoma
Source: JNCI Cancer Spectr. 2023 May 18;7(3):pkad036. doi: 10.1093/jncics/pkad036 (PMC10276895; doi:10.1093/jncics/pkad036)
Supplement: pkad036_Supplementary_Data [file pkad036_supplementary_data.zip › JNCI-CS RR Supplementary Material.v2 PDF.pdf]

Supplementary Material

Supplementary Methods

Study population

The institutional review board of Yale School of Medicine determined this study was exempt including waivers of HIPAA authorization and informed consent due to the use of secondary data for research purposes. This study followed the Strengthening the Reporting of Observational Studies in Epidemiology (STROBE) reporting guideline for cohort studies.

We conducted a retrospective cohort study of Medicare beneficiaries over age 65 diagnosed with mRCC from 2015 through 2019, who were enrolled in fee-for-service Medicare Parts A, B, and D from 1 year prior through 1 year after presumed diagnosis or until death. Patients enrolled in Medicare Advantage plans were excluded as we did not have access to their claims data. We note that Medicare beneficiaries with Medicare Advantage coverage and those who are enrolled due to disability or end-stage renal disease have documented higher membership of NHB and Hispanic race and ethnicity. Thus, the current study population may be relatively overrepresented with individuals of NHW race and ethnicity.

We identified the cohort using diagnosis codes for primary kidney and secondary malignancies, requiring a first position diagnosis for kidney cancer (International Classification for Disease Ninth Revision-(ICD-9) 189.0 or ICD-10 C64.*), as well as two claims with distinct service dates and any position diagnosis of secondary malignancy (ICD-9 196.*, 197.*, 198.*; ICD-10 C77.*, C78.*, C79.*) within 365 days, starting no earlier than 60 days prior to the earliest claim indicating primary kidney cancer. We defined the time of presumed diagnosis as the date of the earliest claim indicating secondary malignancy. This type of approach has been used by prior authors to identify metastatic index date for individuals reported to cancer registries with earlier stage kidney cancer.(1,2) We additionally excluded beneficiaries with > 1 day of claims indicating another primary malignancy (except non-melanoma skin cancers) during the 365 days prior to the index date. These other primary malignancies included claims with the following diagnosis codes: ICD-9: 140-165, 170-176, 179, 180-195, 199-208, 189.1-189.9; ICD-10: C00-C08, C10-C11, C13-C23, C25-C26, C30-C34, C37-C41, C44, C46, C48-C50, C53-C62, C65-C76, C80-C88, C90-C95.

We queried claims from 2014-2020, identifying receipt of IOs, OAAs, or other systemic therapies in the 2 months before through 1 year after diagnosis. The specific therapeutic agents considered in the analysis are detailed in **Supplementary Table 1**. Patients that received both IOs and OAAs were categorized as IO if both therapies were started within 60 days (n = 519 patients, comprising 3.4% of the total cohort). Otherwise, patients were categorized by the first therapy received. While this approach may not be able to capture specific agents received during an inpatient stay due to the structure of Inpatient Medicare claims, the agents of focus are given for more than one dose over time so that even if we do not capture the initial dose, we would capture a subsequent treatment with that type of agent. Race-ethnicity was categorized as American Indian/Native Alaskan/Other (AINAO; combined due to small sample sizes), Asian/Pacific Islander (API), Hispanic, non-Hispanic Black (NHB), or non-Hispanic White (NHW). The race-ethnicity data were from the Research Triangle Institute Race Code variable within the Medicare Beneficiary Summary File.(3)

Statistical analysis

We examined the use of IOs, OAA, or other systemic therapies as initial treatment for mRCC from 2015-2019, further stratifying by race-ethnicity. Treatment rates are reported without adjustment for covariates. Due to small sample sizes for patients of API, Hispanic, and AINAO race and ethnicity, these categories were further aggregated for reporting of yearly treatment rates. In all other contexts, race and ethnicity was categorized into AINAO, API, NHB, Hispanic, or NHW. Data points with $N < 11$ were masked and set to 11. We used Chi-squared tests to compare treatment rates.

We constructed logistic regression models with the initial systemic therapy received (any or type of treatment [multinomial logistic regression]) as the dependent variable, and included year of diagnosis, race and ethnicity, sex, age at diagnosis, Elixhauser comorbidity score(4), Kim claims-based frailty index(5), metropolitan residential status, Medicare/Medicaid dual-eligibility, and Part D Low Income Subsidy eligibility as independent variables. Continuous variables (namely age) were converted to categorical variables to aid in clinical interpretation using commonly accepted thresholds. In accordance with the Stata best practices, we reported the adjusted odds ratios (aORs) for each predictor in relation to receipt of any systemic therapy, and adjusted relative risk ratios (aRRRs) for each predictor in relation to OAA or IO receipt for the

multinomial logistic regression. Patients who did not receive systemic therapy within the predetermined timeframe were the reference group in the models. All patients included in the study sample were required to survive at least 30 days after the index date. Overall, the percentage of surviving patients at least 1 year after the index date was high across treatment categories: 52% no treatment, 64% OAA, 63% IO, and 54% Other systemic therapy. We used SAS 9.4 for data management, Stata 14.2 for statistical analysis, and R 4.0.2 for creating figures.

Supplementary Table 1:
Treatment codes included in analysis.

Supplementary Table 2:
Demographics of the final study cohort.

Supplementary Figure 1

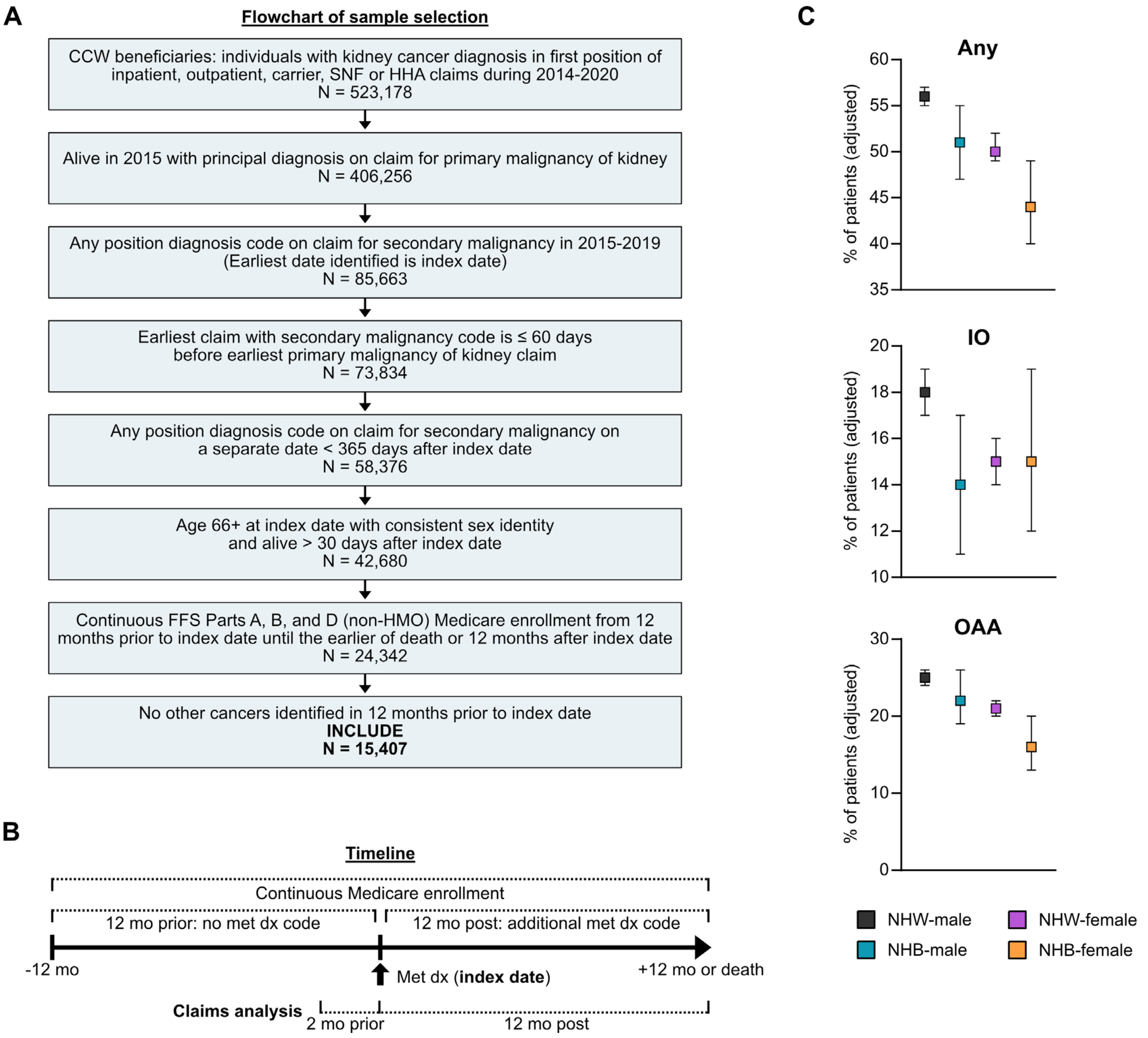

Supplementary Figure 1: Study design and intersectionality analysis

A. Flowchart of sample selection

B. Timeline of sample selection and claims analysis.

C. Predicted probability of systemic therapy receipt in different patient groups, estimated from logistic regression models with multiple sociodemographic factors and an additional interaction term between race-ethnicity and sex. Data are shown as predicted treatment probabilities with 95% confidence intervals shown.

CCW, Chronic Conditions Warehouse; SNF, skilled nursing facility; HHA, home health agency; FFS, fee-for-service; HMO, heath maintenance organization. NHW: Non-Hispanic White. NHB: Non-Hispanic Black.

Supplementary References

1. Wilson LE, Spees L, Pritchard J, et al. Real-World Utilization of Oral Anticancer Agents and Related Costs in Older Adults with Metastatic Renal Cell Carcinoma in the United States. *Kidney Cancer*. 5(3):115-127. doi:10.3233/KCA-210119

2. Spees LP, Wheeler SB, Jackson BE, et al. Provider- and patient-level predictors of oral anticancer agent initiation and adherence in patients with metastatic renal cell carcinoma. *Cancer Med*. 2021;10(19):6653-6665. doi:10.1002/cam4.4201

3. Eicheldinger C, Bonito A. More accurate racial and ethnic codes for Medicare administrative data. *Health Care Financ Rev*. 2008;29(3):27-42.

4. Elixhauser A, Steiner C, Harris DR, Coffey RM. Comorbidity measures for use with administrative data. *Med Care*. 1998;36(1):8-27. doi:10.1097/00005650-199801000-00004

5. Kim DH, Schneeweiss S, Glynn RJ, Lipsitz LA, Rockwood K, Avorn J. Measuring Frailty in Medicare Data: Development and Validation of a Claims-Based Frailty Index. *J Gerontol A Biol Sci Med Sci*. 2018;73(7):980-987. doi:10.1093/gerona/glx229
